# Development and validation of a predictive model for new-onset atrial fibrillation in sepsis based on clinical risk factors

**DOI:** 10.3389/fcvm.2022.968615

**Published:** 2022-08-23

**Authors:** Zhuanyun Li, Ming Pang, Yongkai Li, Yaling Yu, Tianfeng Peng, Zhenghao Hu, Ruijie Niu, Jiming Li, Xiaorong Wang

**Affiliations:** ^1^Department of Emergency Medicine, Union Hospital, Tongji Medical College, Huazhong University of Science and Technology, Wuhan, China; ^2^Department of Neurophysiology, Cangzhou Hospital of Integrated Traditional Chinese Medicine and Western Medicine, Cangzhou, China; ^3^Department of Emergency Medicine, The First Affiliated Hospital, Xinjiang Medical University, Ürümqi, China; ^4^Department of Respiratory and Critical Care Medicine, Union Hospital, Tongji Medical College, Huazhong University of Science and Technology, Wuhan, China

**Keywords:** new-onset atrial fibrillation, nomogram, predictive model, sepsis, SOFA score

## Abstract

**Objective:**

New-onset atrial fibrillation (NOAF) is a common complication and one of the primary causes of increased mortality in critically ill adults. Since early assessment of the risk of developing NOAF is difficult, it is critical to establish predictive tools to identify the risk of NOAF.

**Methods:**

We retrospectively enrolled 1,568 septic patients treated at Wuhan Union Hospital (Wuhan, China) as a training cohort. For external validation of the model, 924 patients with sepsis were recruited as a validation cohort at the First Affiliated Hospital of Xinjiang Medical University (Urumqi, China). Least absolute shrinkage and selection operator (LASSO) regression and multivariate logistic regression analyses were used to screen predictors. The area under the ROC curve (AUC), calibration curve, and decision curve were used to assess the value of the predictive model in NOAF.

**Results:**

A total of 2,492 patients with sepsis (1,592 (63.88%) male; mean [SD] age, 59.47 [16.42] years) were enrolled in this study. Age (OR: 1.022, 1.009–1.035), international normalized ratio (OR: 1.837, 1.270–2.656), fibrinogen (OR: 1.535, 1.232–1.914), C-reaction protein (OR: 1.011, 1.008–1.014), sequential organ failure assessment score (OR: 1.306, 1.247–1.368), congestive heart failure (OR: 1.714, 1.126–2.608), and dopamine use (OR: 1.876, 1.227–2.874) were used as risk variables to develop the nomogram model. The AUCs of the nomogram model were 0.861 (95% CI, 0.830–0.892) and 0.845 (95% CI, 0.804–0.886) in the internal and external validation, respectively. The clinical prediction model showed excellent calibration and higher net clinical benefit. Moreover, the predictive performance of the model correlated with the severity of sepsis, with higher predictive performance for patients in septic shock than for other patients.

**Conclusion:**

The nomogram model can be used as a reliable and simple predictive tool for the early identification of NOAF in patients with sepsis, which will provide practical information for individualized treatment decisions.

## Introduction

Atrial fibrillation (AF) is one of the common types of arrhythmia with a high prevalence, and it is involved in the development of heart failure, stroke, myocardial infarction, and death (1–3). In the intensive care unit (ICU), approximately 10–15% of patients in critical illness may develop new-onset atrial fibrillation (NOAF) (4, 5). NOAF signals the criticality of the disease and a possible factor for adverse outcomes (4, 6). Furthermore, NOAF increases the cost of treatment (cost ratio: 1.09, 1.02–1.20), length of stay in the ICU (median IQR: 6.7, 4.8–12.1), and the mortality rate (OR: 1.28, 1.09–1.36) of patients (7, 8). Although the prognosis for patients with NOAF is poor, there is no early and effective tool to predict NOAF.

Unlike AF in non-critical patients, the pathogenesis of NOAF in sepsis may be more complex. Inflammatory factors increase CD31 expression in cardiomyocytes (9) and inhibit K^+^ channel currents, enhance Na^+^/Ca^2+^ exchange, prolong action potential duration, and increase the risk of arrhythmogenesis (10). At the same time, increased body temperature due to infection affects the effect of sodium channel blockers on Na^+^ currents, decreases the efficacy of some antiarrhythmic drugs, and increases patient mortality (11). Previous studies have suggested various risk factors for NOAF, such as age, vasopressor selection, inflammatory indicators, etc (6, 12, 13). In addition, stress on the myocardium is an important factor, such as takotsubo syndrome. Increased ventricular load causes stretching of the cell membrane and changes in ion channels and electrical activity in cardiac myocytes, causing mechanical-electrical feedback and inducing arrhythmias (14, 15). However, a set of practical and convenient prediction models of NOAF have not been developed after various risk factors have been put forward. The application value of dispersed risk factors in clinical work is limited.

We believe that early identification of people at high risk for NOAF in sepsis is the most appropriate investment to save lives and alleviate the strain on healthcare resources. Firstly, we mainly conducted a retrospective analysis of previous case data to determine the risk factors of NOAF in patients with sepsis. Secondly, we established a predictive model of NOAF based on risk factors. Furthermore, we evaluate this predictive model’s validity and application value to inform decisions for individualized treatment.

## Materials and methods

### Study design and setting

**FIGURE 1:**
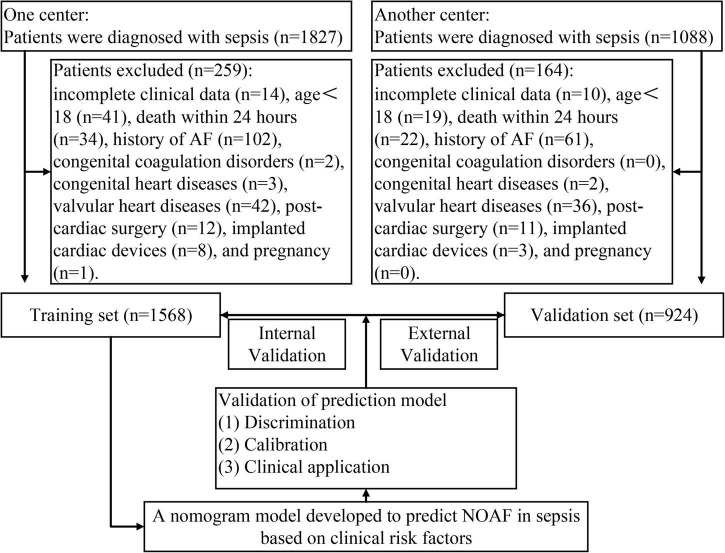
The flow diagram of developing and validating the prediction model.

This project retrospectively reviewed 1,827 patients diagnosed with sepsis at Union Hospital, Tongji Medical College, Huazhong University of Science and Technology between January 2015 and December 2019. Based on the inclusion and exclusion criteria, 1,568 adults with sepsis were ultimately enrolled in the training cohort (994 (63.39%) male; mean [SD] age, 59.26 [6.23] years). From January 2015 to December 2019, an independent validation cohort of 924 patients (598 (64.72%) male; mean [SD] age, 59.84 [16.72] years) was screened from 1,088 patients using the same criteria at The First Affiliated Hospital of Xinjiang Medical University. The flow diagram for developing and validating the prediction model was illustrated in Figure 1. The current project followed the principle of the Declaration of Helsinki. The work was approved by the Ethics Committee of the Union Hospital of Tongji Medical College, Huazhong University of Science and Technology, and written informed consent was not required (No.2021-0956).

### Participants and data collection

The diagnostic criteria of sepsis are based on the 2016 edition of Sepsis-3. The diagnostic criteria are as follows: (i) patients with confirmed or suspected infection; (ii) SOFA score ≥ 2 (16). The determination of NOAF was based on the electrocardiogram report in the case data and the hourly rhythm record in the nursing record. NOAF was defined as (i) no history of AF; (ii) AF lasting > 1 h; or (iii) paroxysmal AF or atrial flutter intervened with pharmacological therapy or electrical resuscitation (6). Patients with the following conditions were excluded: incomplete clinical data, age < 18, death within 24 h, history of AF, congenital coagulation disorders, congenital heart diseases, valvular heart diseases, post-cardiac surgery, implanted cardiac devices, and pregnancy.

The following clinical data were collected within 24 h of patient admission: gender, age, body mass index (BMI), pre-admission comorbidities, coagulation, liver and renal function, B-type natriuretic peptide (BNP), procalcitonin, international normalized ratio (INR), cardiac troponin I, C-reaction protein (CRP), sequential organ failure assessment (SOFA) score, site of infection, and pathogens, etc. If a variable reported more than one value in the first 24 h, the worst was selected for analysis.

### Outcomes

The primary observation was the incidence of NOAF in patients with sepsis. Secondary observations were the length of stay in the hospital, in-hospital mortality, length of ICU stay, and readmission to the ICU during hospitalization.

### Statistical analysis

The baseline information of the study population was analyzed by descriptive statistics. The Kolmogorov–Smirnov test accomplished the normality distribution of continuous variables. Normally distributed continuous variables were expressed as mean and standard deviation and vice versa as median and interquartile range. For categorical variables, frequencies and percentages are the best way to represent them. The least absolute shrinkage and selection operator (LASSO) is a powerful method for regression with high-dimensional predictors. Our study used the LASSO binary logistic regression model for risk factor selection, and factors with non-zero coefficients were selected. Multivariate logistic regression analysis assessed the association between risk factors and NOAF and created a nomogram based on selected variables.

The accuracy of the nomogram model can be performed by internal and external validation. The area under the ROC curve (AUC) is used to assess the model’s discrimination. Calibration plots are more meaningful for evaluating the degree of model fit, which assesses how close the actual results of each nomogram are to the predicted results (17). Decision curve analysis (DCA) shows the standardized net benefit relative to the risk threshold probability and is used to assess the clinical utility of the model (18). The clinical impact curves show the number of high-risk and true-positive patients at different threshold probabilities. In addition, Kaplan–Meier curves and log-rank tests were used in the survival analysis.

Statistical analysis was conducted with SPSS (IBM SPSS Statistics 26.0, SPSS Inc., Chicago, IL, United States) and R language (version 4.1.3).^1^ The R packages used in our study were displayed in Supplementary Table 1. All statistical tests were two-sided, and statistical significance was set at 0.05.

## Results

### Demographic and baseline characteristics

In this study, 2,492 patients with sepsis were enrolled, of whom 269 (10.8%) had NOAF. The median age was 59, ranging from 18 to 94 years old. Male patients comprised 63.9% of the total. The demographic data between the training and validation cohorts were described (Table 1). The variables were well balanced between the two cohorts, except for the prevalence of chronic obstructive pulmonary disease, the rate of skin soft tissue infections, and albumin levels. No statistical differences were observed in the training cohort for the three variables mentioned above when compared between the NOAF and non-NOAF groups (Supplementary Table 2).

**TABLE 1:** Comparison of characteristics between the training and validation cohorts.

Variables	All patients (*n* = 2,492)	Training cohort (*n* = 1,568)	Validation cohort (*n* = 924)	*P-*value
**Gender, *n* (%)**				0.506
Male^§^	1,592 (63.9)	994 (63.4)	598 (64.7)	
Female^§^	900 (36.1)	574 (36.6)	326 (35.3)	
Age (years)*^†^*	59.47 (16.42)	59.26 (16.23)	59.84 (16.72)	0.392
**Physiological data on admission**				
Heart rates (beats/min)*^†^*	105.13 (10.25)	105.38 (10.48)	104.70 (9.83)	0.111
MAP (mm Hg)*^†^*	96.40 (7.01)	96.60 (6.11)	96.08 (8.31)	0.074
BMI (kg/m^2^)*^†^*	22.06 (1.89)	22.12 (1.87)	21.98 (1.91)	0.091
**Comorbidity, *n* (%)**				
Hypertension^§^	449 (18.0)	274 (17.5)	175 (18.9)	0.358
Coronary artery disease^§^	234 (9.4)	136 (8.7)	98 (10.6)	0.110
Congestive heart failure^§^	559 (22.4)	364 (23.2)	195 (21.1)	0.223
Diabetes mellitus^§^	337 (13.5)	227 (14.5)	110 (11.9)	0.070
COPD^§^	186 (7.5)	130 (8.3)	56 (6.1)	0.041
Hyperlipidemia^§^	556 (22.3)	345 (22.0)	211 (22.8)	0.630
Stroke^§^	183 (7.3)	124 (7.9)	59 (6.4)	0.159
Hepatic insufficiency^§^	199 (8.0)	123 (7.8)	76 (8.2)	0.735
Renal insufficiency^§^	273 (11.0)	158 (10.1)	115 (12.4)	0.067
Cancer^§^	70 (2.8)	47 (3.0)	23 (2.5)	0.458
**Infection site, *n* (%)**				
Pulmonary^§^	1318 (52.9)	837 (53.4)	481 (52.1)	0.523
Intra-abdominal^§^	541 (21.7)	322 (20.5)	219 (23.7)	0.064
Genitourinary^§^	366 (14.7)	233 (14.9)	133 (14.4)	0.751
Skin and soft tissue^§^	108 (4.3)	80 (5.1)	28 (3.0)	0.014
Blood stream^§^	344 (13.8)	218 (13.9)	126 (13.6)	0.852
**Type of pathogen, *n* (%)**				
Bacteria^§^	2319 (93.1)	1459 (93)	860 (93.1)	0.981
Fungi^§^	200 (8.0)	134 (8.5)	66 (7.1)	0.213
**Severity on admission**				
SOFA score*	5.00 (3.00–7.00)	5.00 (3.00–7.00)	5.00 (2.00, 7.00)	0.093
APACHE II score*	15.00 (10.00–18.00)	15.00 (10.00–18.00)	14.00 (9.00–18.00)	0.204
SAPS II score*	42.00 (36.00–46.00)	42.00 (36.00–46.00)	42.00 (36.00–46.00)	0.424
**Laboratory tests**				
White blood cell count (× 10^9^/L)*	13.40 (12.00–14.60)	13.40 (12.30–14.40)	13.30 (10.40–15.20)	0.204
Hemoglobin (g/L)*	114.00 (111.00–117.00)	114.00 (111.00–117.00)	114.00 (111.00–117.00)	0.299
Platelet count (×10^9^/L)*	156.0 (98.00–164.00)	155.0 (98.00–164.00)	156.0 (98.00–164.75)	0.291
Platelet distribution width (%)*	16.10 (15.40–16.80)	16.00 (15.40–16.70)	16.10 (15.40–16.80)	0.277
Serum creatinine (μmol/L)*	80.44 (73.23–86.67)	80.28 (72.94–86.63)	80.91 (73.58–86.70)	0.154
Blood urea nitrogen (mmol/L)*	7.20 (5.70–8.40)	7.10 (5.70–8.40)	7.20 (5.70–8.48)	0.989
ALT (U/L)*	35.00 (24.00–47.00)	35.00 (23.25–47.00)	35.00 (24.00–48.00)	0.633
Bilirubin (μmol/L)*	25.03 (21.80–28.49)	25.10 (21.88–28.70)	24.89 (21.76–28.26)	0.190
Albumin (g/L)*	39.84 (34.73–44.82)	40.12 (35.11–44.91)	39.36 (34.07–44.57)	0.021
Cardiac troponin I (ng/mL)*	0.05 (0.04–0.06)	0.05 (0.04–0.06)	0.05 (0.04–0.06)	0.529
BNP (pg/mL)*	94.42 (80.90–108.93)	94.50 (81.01–108.48)	94.38 (80.40–109.85)	0.562
APTT (s)*	35.20 (31.62–38.70)	35.20 (31.70–38.70)	35.30 (31.60–38.88)	0.771
PT (s)*	15.20 (13.70–17.40)	15.20 (13.70–17.40)	15.10 (13.60–17.30)	0.096
INR*	1.28 (1.10–1.72)	1.27 (1.10–1.70)	1.29 (1.10–1.74)	0.376
Fibrinogen (g/L)*	4.06 (3.69–4.44)	4.07 (3.70–4.44)	4.01 (3.66–4.43)	0.123
D-dimer (mg/L)*	2.92 (1.62–6.39)	2.98 (1.65–6.31)	2.81 (1.55–6.40)	0.642
Lactic acid (mmol/L)*	4.40 (3.69–5.11)	4.40 (3.71–5.12)	4.37 (3.67–5.09)	0.411
Procalcitonin (μg/L)*	3.03 (2.70–3.40)	3.03 (2.69–3.39)	3.05 (2.70–3.40)	0.639
CRP (mg/L)*	46.00 (17.92–89.36)	45.30 (18.04–88.30)	47.40 (17.45–90.59)	0.790
**Treatment measures, *n* (%)**				
Corticosteroid use^§^	583 (23.4)	366 (23.3)	217 (23.5)	0.935
Epinephrine use^§^	136 (5.5)	96 (6.1)	40 (4.3)	0.057
Norepinephrine use^§^	578 (23.2)	383 (24.4)	195 (21.1)	0.058
Dopamine use^§^	538 (21.6)	322 (20.5)	216 (23.4)	0.096
**Outcome, *n* (%)**				
New-onset atrial fibrillation^§^	269 (10.8)	167 (10.7)	102 (11.0)	0.763

^†^Normally distributed continuous variables are presented as means with standard deviations and analyzed by Student’ s *t*-test.

*Non-normally distributed continuous variables are presented as medians with interquartile ranges and analyzed by non-parametric test.

^§^Categorical variables are presented as frequencies with percentages and analyzed by Chi-square test or Fisher’ s exact test.

MAP, mean arterial pressure; BMI, body mass index; COPD, chronic obstructive pulmonary disease; SOFA score, sequential organ failure assessment score; APACHE II score, acute physiology and chronic health evaluation II score; SAPS II, simplified acute physiology score II; ALT, alanine aminotransferase; BNP, B-type natriuretic peptide; APTT, activeated partial thromboplasting time; PT, prothrombin time; INR, international normalized ratio; CRP, C-reaction protein.

### The construction of predictive model based on risk factors

**FIGURE 2:**
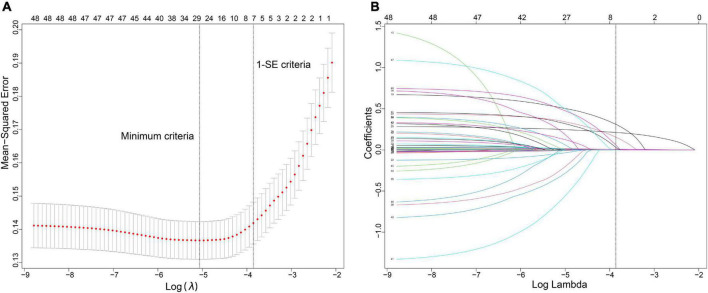
Variable selection using the least absolute shrinkage and selection operator (LASSO) binary logistic regression model. **(A)** The tuning parameter (λ) in the LASSO model was selected for 10-fold cross-validation by the minimum criteria. The dotted vertical lines were drawn at the best values using the minimum criteria and 1 standard error of the minimum criteria (the 1-SE criteria). A λ-value of 0.021, with log (λ), –3.855 was chosen (1-SE criteria) according to 10-fold cross-validation. **(B)** LASSO coefficient curves of the 48 variables. A coefficient profile plot was produced against the log (λ) sequence. Vertical line was drawn at the value selected using 10-fold cross-validation, where optimal λ resulted in 7 non-zero coefficients.

**FIGURE 3:**
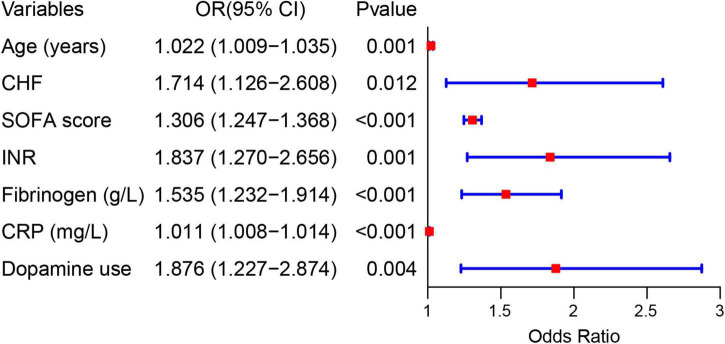
Forest plot showing the relationship between risk factors and the development of new-onset atrial fibrillation in patients with sepsis.

**FIGURE 4:**
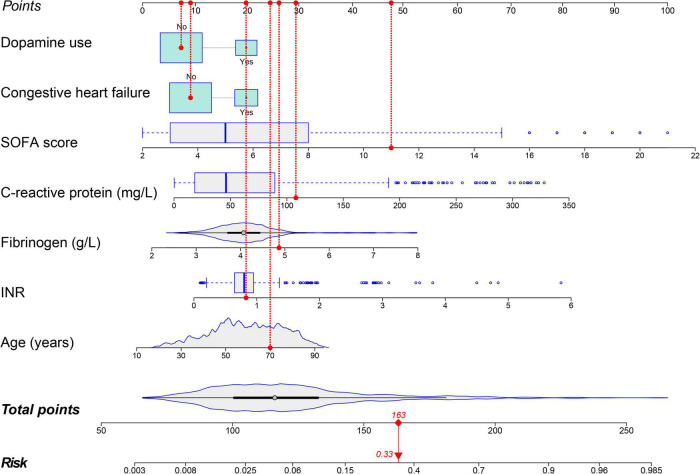
Nomogram for predicting the risk of new-onset atrial fibrillation in patients with sepsis. A 70-year-old patient with sepsis and no history of congestive heart failure. During hospitalization INR was 0.83, fibrinogen was 4.87 g/L, C-reactive protein was 108 mg/L, SOFA score was 11, and dopamine was not used during treatment. This patient had a total score of 163 and a 33.0% risk of developing new-onset atrial fibrillation.

Forty-eight variables in the training cohort of 1,568 patients with sepsis (167 with NOAF) were screened by the LASSO binary logistic regression model, which selected 7 predictors with non-zero coefficients (Figures 2A,B and Supplementary Table 3). After multivariate logistic regression analysis, age, congestive heart failure (CHF), SOFA score, INR, fibrinogen, CRP, and dopamine use were independent risk factors for NOAF (Figure 3). We weighted the regression coefficients of risk factors in multivariate logistic regression and developed a risk score formula to predict NOAF. Risk score = −8.296 + 0.022 (age) + 0.539 (if CHF is positive) + 0.267 (SOFA score) + 0.608 (INR) + 0.429 (fibrinogen) + 0.011 (CRP) + 0.629 (if dopamine is used). Predicted risk = 1/(1 + e^–*riskscore*)^ (Table 2). The nomogram model for predicting the probability of NOAF was developed based on the above risk factors. A true case is presented in Figure 4.

**TABLE 2:** Association between risk factors and new-onset atrial fibrillation in multivariate logistic regression.

Variables	β	OR (95% CI)	*P-*value
Intercept	−8.296		<0.001
Age (years)	0.022	1.022 (1.009–1.035)	0.001
**Comorbidity**			
Congestive heart failure	0.539	1.714 (1.126–2.608)	0.012
**Severity on admission**			
SOFA score	0.267	1.306 (1.247–1.368)	<0.001
**Laboratory tests**			
INR	0.608	1.837 (1.270–2.656)	0.001
Fibrinogen (g/L)	0.429	1.535 (1.232–1.914)	<0.001
CRP (mg/L)	0.011	1.011 (1.008–1.014)	<0.001
**Treatment measures**			
Dopamine use	0.629	1.876 (1.227–2.874)	0.004

SOFA score, sequential organ failure assessment score; INR, international normalized ratio; CRP, C-reaction protein.

### Validation and evaluation of the nomogram

The validation of the nomogram in this study was performed using internal and external validation.

#### Internal validation

**FIGURE 5:**
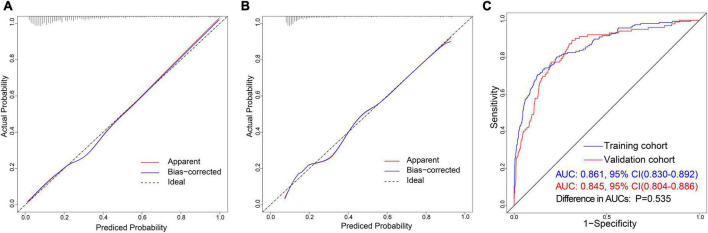
Discrimination and calibration of nomogram prediction models in the training and validation cohorts. **(A)** Calibration plot in the training cohort. **(B)** Calibration plot in the validation cohort. **(C)** ROC curves in both the training and validation cohorts.

The calibration curve of the nomogram is used to show the agreement between the predicted and observed results. The agreement between the two results performs well in the training cohort (Figure 5A). The Hosmer–Lemeshow results indicated no significant difference, which suggested a good fit in the training cohort (Hosmer–Lemeshow χ^2^ = 3.423, *p* = 0.891). The predictive performance of the nomogram was evaluated by the ROC curve, which had an AUC of 0.861 (95% CI, 0.830–0.892) (Figure 5C).

#### Independent validation

We also observed an excellent calibration effect in the validation cohort (Figure 5B) and no statistical difference in the Hosmer-Lemeshow results (Hosmer–Lemeshow χ^2^ = 4.653, *p* = 0.794). Meanwhile, the area under the ROC curve was 0.845 (95% CI, 0.804–0.886) (Supplementary Table 4). There was no statistically significant difference between the AUCs of the two cohorts (*P* = 0.535) (Figure 5C).

#### Predictive performance of different sepsis severity

To test the performance of the prediction model in different sepsis severity, we divided the patients into sepsis group, severe sepsis group and septic shock group. In the training cohort, CRP, dopamine use, the incidence of NOAF, and in-hospital mortality were higher in the septic shock group than in the other groups (Supplementary Table 5). In addition, the predictive performance of the nomogram model improved with increasing disease severity (Supplementary Figure 1A). The AUC in the septic shock group was 0.913 (0.873–0.953), which was significantly higher than that in the sepsis group (AUC: 0.812, 0.755–0.870) and severe sepsis group (AUC: 0.885, 0.830–0.939) (Supplementary Table 7). We obtained the same conclusion in the validation cohort (Supplementary Figure 1B and Supplementary Table 6).

### Clinical usefulness

**FIGURE 6:**
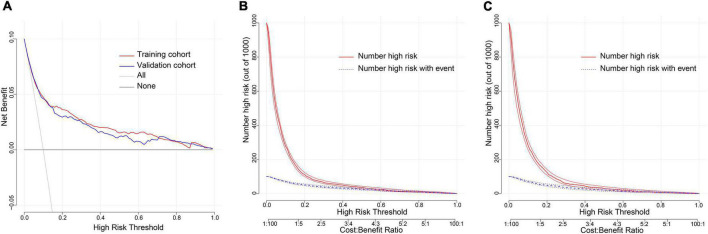
Evaluation of clinical utility of nomogram prediction models in the training and validation cohorts. **(A)** Decision curves in both the training and validation cohorts. **(B)** Clinical impact curve in the training cohort. **(C)** Clinical impact curve in the validation cohort.

Decision curve analysis (DCA) is a method to assess the benefits of a diagnostic test by quantifying the net benefit at different threshold probabilities to determine the clinical usefulness of the nomogram. DCA was applied in this study to assess the nomogram’s clinical utility. Both the training and validation cohorts demonstrated higher clinical net benefit compared to the two thresholds of “no intervention” and “intervention for all” (Figure 6A). The clinical impact curves revealed a convergence between the number of patients considered at high risk of NOAF and those with a NOAF event within this risk threshold (Figures 6B,C). The prediction model had good clinical application.

### Outcomes

**FIGURE 7:**
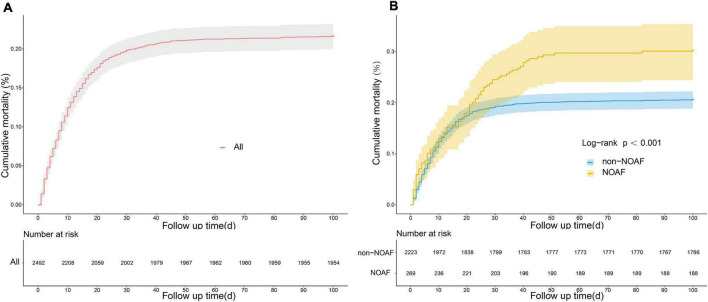
Cumulative mortality in patients with sepsis based on kaplan-meier curves. **(A)** Cumulative mortality in all patients with sepsis. **(B)** Comparison of cumulative mortality between new-onset atrial fibrillation and non-new-onset atrial fibrillation.

A total of 2,492 septic patients were included in this study, of whom 269 septic patients developed NOAF. The length of hospitalization, length of ICU stay, and in-hospital mortality were significantly increased by univariate analysis in the NOAF group versus the non-NOAF group. However, no significant difference was observed in the rate of ICU readmission during hospitalization (Table 3). We found that in-hospital mortality in patients with sepsis increased dramatically in the early stages of hospitalization (Figure 7A). Moreover, in-hospital mortality was significantly higher in the NOAF group than in the non-NOAF group (Figure 7B).

**TABLE 3:** Outcomes in patients with or without new-onset atrial fibrillation.

Outcome	All patients (*n* = 2,492)	non-NOAF (*n* = 2,223)	NOAF (*n* = 269)	χ^2^/Z	*P-*value
Hospital length of stay, median (IQR), d	12.00 (7.00–18.00)	11.00 (7.00–18.00)	13.00 (8.00–21.00)	2.247	0.025
ICU length of stay, median (IQR), d	2.00 (2.00–4.00)	2.00 (2.00–4.00)	4.00 (2.00–6.00)	8.915	<0.001
Readmission to ICU during hospitalization, No. (%)	345 (13.8)	299 (13.5)	46 (17.1)	2.680	0.102
Thromboembolic events, No. (%)	183 (7.3)	153 (6.9)	30 (11.2)	6.430	0.011
In-hospital mortality, No. (%)	538 (21.6)	457 (20.6)	81 (30.1)	12.938	<0.001

## Discussion

Our study developed and validated a predictive model for NOAF using clinical data from 2,492 patients with sepsis at two institutions. We identified age, INR, fibrinogen, CRP, SOFA score, CHF, and dopamine use as independent predictors of NOAF by multivariate logistic regression analyses. We developed a nomogram based on these predictors. After validation by multiple methods, the model showed good calibration, discrimination, and clinical utility.

Investigators have conducted in-depth studies on sepsis to manage patients with NOAF in sepsis better. In a study by Moss TJ et al. that included 8,356 critically ill patients, advanced age and sepsis were noted as significant risk factors for NOAF, yet no predictive models were constructed (19). In the systematic analysis by Wetterslev M’s team, risk factors for NOAF were systematically analyzed and discussed, but no easy and practical prediction model was developed (5). Furthermore, one study developed a risk factor scoring system for NOAF in sepsis, but the scoring system was more complex to operate and had a C statistic of 0.81 (95% CI, 0.79–0.84), with poor predictive performance (6). Therefore, the present study applied the visualized nomogram model to predict NOAF in sepsis, and the model’s predictive performance was better than the studies above, which was more applicable in clinical practice.

Advancing age is one of the prominent risk factors for the development of AF, and epidemiological studies have found a progressive increase in the prevalence of AF with increasing age. With aging, the myocardium will undergo anatomical and electrophysiological changes. The atrial myocardium may lose lateral electrical connections between myofibers, and electrical conduction in the sinoatrial node, atrioventricular node, and atria may be reduced. A multicenter cohort study of a Chinese community population found a prevalence of 0.13% for AF in 51–60 years old (20). The prevalence was 0.11% in the Scottish aged 55–64 (21). In contrast, the mean age of septic patients in this study was 59 years. The prevalence of AF was 10.8%, significantly higher than the prevalence in the community population of the same age. In addition, some studies have shown that gender, BMI, and hypertension were risk factors for the development of AF (6, 12, 19). However, the above variables were not statistically different in this study, which may be related to the different populations included in the study, such as septic patients combined with multi-organ dysfunction. Therefore, NOAF may result from multiple factors.

It is well known that AF contributes to heart failure and vice versa. The pathogenesis of AF is structural remodeling and abnormal electrical activity of the atria (22, 23). The prevalence of AF in patients with congestive heart failure was 26–35%, and its pathogenesis may be caused by intracellular calcium dysregulation, elevated cardiac filling pressures, abnormal autonomic function, and neuroendocrine dysfunction (24). Thus, CHF may provide an “arrhythmogenic substrate” for the development of AF. In this study, CHF was identified as a significant risk factor for NOAF, with a 1.714-fold risk of AF, which was consistent with previous studies (25). However, a meta-analysis proposed that CHF was a significant risk factor for community-associated AF, with a diminished role in patients with sepsis (12). Patients with sepsis often have internal environmental disturbances and multi-organ dysfunction, and the combined effect of multiple factors may diminish the predictive value of CHF.

Our findings indicated that the risk of NOAF during sepsis was driven more by sepsis-related events and therapy, except for non-modifiable factors (age and history of CHF). Currently, more studies suggest that inflammation promotes the development of AF (26, 27). Inflammatory indicators can reduce myocardial contractility by upregulating myocardial nitric oxide synthase and downregulating sarcoplasmic reticulum Ca^2+^ATPase (28). In addition, inflammatory cell infiltration in cardiac myocytes leads to myocardial microabscesses and promotes myocardial fibrosis (29). Some studies have noted an association between leukocyte counts and AF (30). However, more studies focus on CRP as a primary predictor of NOAF (31, 32). CRP could act on monocytes/macrophages, vascular endothelial cells, and smooth muscle cells to secrete pro-inflammatory molecules to induce cardiovascular disease (33). The prevalence of AF was increased during sepsis when CPR was ≥ 70 mg/L (12). In this study, the CRP level in the NOAF group was 67.11 (95%CI, 30.58–110.00) mg/L, which was lower than 70 mg/L but significantly higher than the CRP level in the community population with NOAF (<10 mg/L) (34). The main reason was the greater degree of infection in septic patients compared to the community population. Moreover, the incidence of pulmonary infection was 67.1% in the NOAF group, which was higher than that in the non-NOAF group (*P* < 0.001), the result consistent with the findings of previous studies (35). The specific pathogenesis might be related to cytokine production and secondary myocardial suppression, but confirmation by further studies is needed.

Another indicator of inflammation, IL-6, is a cytokine with multiple biological functions. Not only associated with left ventricular hypertrophy and systolic dysfunction, but it is also a risk factor for the development of AF in patients with coronary artery disease (36). IL-6 increases AF susceptibility by mediating Ca^2+^ handling in cardiomyocytes, leading to RyR2 dysfunction (37). In a study that included 371 patients with coronary artery bypass grafting, IL-6 gene expression levels were higher in the postoperative AF group than in the non-AF group and were independently correlated with postoperative AF (odds ratio: 2.01, 95% CI: 1.15–3.52) (38). Moreover, increased IL-6 levels were also related to an increased risk of death in patients with AF (39, 40). However, the absence of IL-6 data in this study did not allow exploring the relationship between it and AF. We will study the relationship between IL-6 and AF at a later stage.

The SOFA score is widely used in clinical work as an essential criterion for diagnosing sepsis (16). It includes an assessment of dysfunction in six organ systems and a scoring system to assess the severity of disease and prognosis in critically ill patients (41). A prospective study identified the SOFA cardiovascular score as an independent risk factor for NOAF (42). The median SOFA score in the NOAF group was 6 in this study. It was proved to be one of the risk factors predicting NOAF, similar to the findings of the above studies, but we did not compare the scores of each organ system.

Dysfunction of the coagulation system, known as sepsis-associated coagulopathy, also occurs during sepsis. Sepsis-associated coagulopathy consists of a prolonged INR and a reduced platelet count, which was related to 28-day mortality in septic patients and was one way to assess disease severity (43, 44). In a retrospective study of sepsis, coagulopathy within 24 h of admission was an independent risk factor for AF, with an INR of 1.5 (95%CI, 1.2–2.2) in the AF group (45). The INR was 1.46 (95%CI, 1.20–3.26) in this study, consistent with the above findings. The INR values were higher than those in the non-NOAF group. We also found a significant decrease in platelet count, a higher incidence of sepsis-associated coagulopathy, and higher disease severity in the NOAF group. Furthermore, fibrinogen was also related to the development of AF in this study. Fibrinogen levels were significantly higher in septic patients, and fibrinogen production was more than three times higher than in non-septic patients (46). Fibrinogen was elevated in permanent and paroxysmal AF in a prospective study (47). In addition, the fibrinogen level was 3.33 ± 0.9 in the idiopathic AF group, which was higher than in the control group (*P* < 0.05) (48). These results were consistent with our finding that fibrinogen was associated with AF development. Therefore, we should not ignore the coagulation indicators as a risk factor.

Sometimes sepsis-related therapy can also be a risk factor for the development of AF. Dopamine, a vasoactive drug, is widely used in patients with sepsis. However, the cardiac adverse events with dopamine use have also attracted more attention (49). In patients undergoing coronary artery bypass graft surgery, the incidence of AF was 23.3% with postoperative dopamine use, higher than the 14.1% rate in the non-dopamine group (50). In a meta-analysis that included 2,768 patients in septic shock, the dopamine use resulted in a higher incidence of arrhythmic events and patient mortality than norepinephrine (51); the same conclusion was obtained in 1,679 patients in shock (52). Our study further confirmed dopamine as a risk factor for NOAF. Hemodynamic instability often accompanies patients with sepsis and requires maintenance therapy with vasoactive drugs. Dopamine may cause positive inotropic and positive chronotropic effects (increased contractility and rate) by activating β1-adrenergic receptors in the heart (53). The incidence of arrhythmias, most commonly in AF, is increased at high doses (>10 μg kg^–1^ min^–1^). Therefore, more caution is needed in using dopamine when treating patients with sepsis.

Currently, much more studies are focusing on genomics (54) and extracellular vesicles (55) in the development of AF. As more relevant studies are explored, more new therapeutic targets for AF will be identified, which will help improve the prevention and management of AF. This study also has some limitations. First, it was a non-randomized retrospective analysis and may have potential comparison biases such as sample selection and patient inclusion bias. Second, although the study found a higher mortality rate in the NOAF group than in the non-NOAF group, it does not equate to a causal relationship between NOAF and sepsis prognosis, which needs further confirmation by prospective studies with large samples. Finally, relevant results from advanced genomics and cardiac magnetic resonance imaging were not included. However, our findings are expected to combine with genomics or other markers to enable AF prediction models to achieve higher predictive power.

## Conclusion

In this study, we developed and validated a nomogram model to predict the prevalence of NOAF during sepsis. The model achieves individualized prediction of NOAF during hospitalization in patients with sepsis and offers the possibility of early intervention and reduction of the prevalence of AF.

## Data availability statement

The original contributions presented in this study are included in the article/Supplementary material, further inquiries can be directed to the corresponding authors.

## Ethics statement

The studies involving human participants were reviewed and approved by the Ethics Committee of the Union Hospital of Tongji Medical College, the Huazhong University of Science and Technology (No. 2021-0956). Written informed consent for participation was not required for this study in accordance with the national legislation and the institutional requirements.

## Author contributions

ZL, MP, XW, and JL: design, drafting, and revision of the manuscript. ZL, YL, YY, TP, ZH, RN, and JL: collection of clinical data. ZL, MP, and YL: statistical analysis. XW and JL: guidance on the research process and revision of the article. All authors contributed to the manuscript.

## Footnotes

1
www.R-project.org/
