# Supplementary material for: Development and validation of a predictive model for new-onset atrial fibrillation in sepsis based on clinical risk factors
Source: Front Cardiovasc Med. 2022 Aug 23;9:968615. doi: 10.3389/fcvm.2022.968615 (PMC9447992; doi:10.3389/fcvm.2022.968615)
Supplement: Supplementary file 1 [file Data_Sheet_1.docx]

**Supplementary Online Content**

**Supplementary Table 1.** The R packages used in our study

**Supplementary Table 2.** Comparison of baseline information between the new-onset atrial fibrillation group and the non-new-onset atrial fibrillation group in the training cohort

**Supplementary Table 3.** Coefficients and lambda.1-SE value of the LASSO regression

**Supplementary Table 4.** Diagnostic performance of the nomogram model for new-onset atrial fibrillation in the training and validation cohorts

**Supplementary Table 5.** Comparison of different severity of sepsis in the training cohort

**Supplementary Table 6.** Comparison of different severity of sepsis in the validation cohort

**Supplementary Table 7.** Comparison of AUC results of nomogram model in sepsis of different severity

**Supplementary Figure 1.** Comparison of the predictive performance of nomogram model in sepsis of different severity

**Supplementary Table 1.** The R packages used in our study.

| **Function** | **Packages** |
| --- | --- |
| Least absolute shrinkage and selection operator (LASSO) regression | “glmnet” and “car” packages; |
| Forest plot | “forestplot” package; |
| Nomograms and calibration curves | “rms” and “Hmisc” packages; |
| Receiver operating characteristic curve | “pROC”, “ROCR” and “gplots” packages |
| Decision curve analysis and clinical impact curve | “rmda” package; |
| Kaplan-Meier curves | “survival”, “dplyr” and “survminer” packages |

**Supplementary Table 2.** Comparison of baseline information between the new-onset atrial fibrillation group and the non-new-onset atrial fibrillation group in the training cohort.

| **Variables** | **All patients**  **（*n*=1568）** | **non-NOAF（*n*=1401）** | **NOAF（*n*=167）** | ***P* Value** |
| --- | --- | --- | --- | --- |
| **Gender, *n* (%)** |  |  |  | 0.132 |
| Male^§^ | 994(63.4) | 897(64.0) | 97(58.1) |  |
| Female^§^ | 574(36.6) | 504(36.0) | 70(41.9) |  |
| Age (years)*^†^* | 59.26±16.23 | 58.48±16.35 | 65.80±13.65 | ＜0.001 |
| Heart rates (beats/min)*^†^* | 105.38±10.48 | 105.14±10.43 | 107.38±10.76 | 0.009 |
| MAP (mm Hg)*^†^* | 96.60±6.11 | 96.47±6.15 | 97.66±6.64 | 0.017 |
| BMI (kg/m^2^)*^†^* | 22.12±1.87 | 22.11±1.85 | 22.16±2.04 | 0.768 |
| **Comorbidity, *n* (%)** |  |  |  |  |
| Hypertension^§^ | 274(17.5) | 231(16.5) | 43(25.7) | 0.003 |
| Coronary artery disease^§^ | 136(8.7) | 116(8.3) | 20(12.0) | 0.109 |
| Congestive heart failure^§^ | 364(23.2) | 307(21.9) | 57(34.1) | ＜0.001 |
| Diabetes mellitus^§^ | 227(14.5) | 201(14.3) | 26(15.6) | 0.671 |
| COPD^§^ | 130(8.3) | 113(8.1) | 17(10.2) | 0.349 |
| Hyperlipidemia^§^ | 345(22.0) | 294(21.0) | 51(30.5) | 0.005 |
| Stroke^§^ | 124(7.9) | 112(8.0) | 12(7.2) | 0.714 |
| Hepatic insufficiency^§^ | 123(7.8) | 110(7.9) | 13(7.8) | 0.976 |
| Renal insufficiency^§^ | 158(10.1) | 136(9.7) | 22(13.2) | 0.160 |
| Cancer^§^ | 47(3.0) | 41(2.9) | 6(3.6) | 0.633 |
| **Infection site, *n* (%)** |  |  |  |  |
| Pulmonary^§^ | 837(53.4) | 725(51.7) | 112(67.1) | ＜0.001 |
| Intra-abdominal^§^ | 322(20.5) | 274(19.6) | 48(28.7) | 0.005 |
| Genitourinary^§^ | 233(14.9) | 204(14.6) | 29(17.4) | 0.336 |
| Skin and soft tissue^§^ | 80(5.1) | 74(5.3) | 6(3.6) | 0.348 |
| Blood stream^§^ | 218(13.9) | 191(13.6) | 27(16.2) | 0.371 |
| **Type of pathogen, *n* (%)** |  |  |  |  |
| Bacteria^§^ | 1459(93.0) | 1306(93.2) | 153(91.6) | 0.442 |
| Fungi^§^ | 134(8.5) | 105(7.5) | 29(17.4) | ＜0.001 |
| **Severity on admission** |  |  |  |  |
| SOFA score^*^ | 5.00(3.00-7.00) | 5.00(3.00-7.00) | 6.00(4.00-9.00) | ＜0.001 |
| APACHE Ⅱ score^*^ | 15.00(10.00-18.00) | 15.00(10.00-18.00) | 16.00(13.00-19.00) | ＜0.001 |
| SAPS Ⅱ score^*^ | 42.00(36.00-46.00) | 41.00(36.00-46.00) | 42.00(38.00-50.00) | 0.001 |
| **Heart function** |  |  |  |  |
| Ejection fraction | 44.50(41.06-48.00) | 45.19(41.64-48.44) | 40.77(38.58-42.67) | ＜0.001 |
| **Laboratory tests** |  |  |  |  |
| White blood cell count (×10^9^/L)^*^ | 13.40(12.30-14.40) | 13.40(12.30-14.40) | 13.70(12.50-14.80) | 0.004 |
| Hemoglobin (g/L)^*^ | 114.00(111.00-117.00) | 114.00(111.00-117.00) | 114.00(111.00-117.00) | 0.378 |
| Platelet count (×10^9^/L)^*^ | 155.0(98.00-164.00) | 156.0(98.00-165.00) | 152.0(98.00-161.00) | 0.005 |
| Platelet distribution width (%)^*^ | 16.00(15.40-16.70) | 16.00(15.40-16.70) | 16.10(15.50-16.90) | 0.207 |
| Serum creatinine (μmol/L)^*^ | 80.28(72.94-86.63) | 80.37(72.85-86.75) | 79.93(74.05-85.67) | 0.800 |
| Blood urea nitrogen (mmol/L)^*^ | 7.10(5.70-8.40) | 7.10(5.70-8.40) | 7.40(5.90-8.50) | 0.265 |
| ALT (U/L)^*^ | 35.00(23.25-47.00) | 35.00(23.00-47.00) | 37.00(24.00-46.00) | 0.773 |
| Bilirubin (μmol/L)^*^ | 25.10(21.88-28.70) | 25.23(21.99-28.72) | 24.10(21.26-28.59) | 0.220 |
| Albumin (g/L)^*^ | 40.12(35.11-44.91) | 40.19(35.44-44.93) | 39.20(33.70-44.51) | 0.094 |
| Cardiac troponin I (ng/mL)^*^ | 0.05(0.04-0.06) | 0.05(0.04-0.06) | 0.05(0.04-0.06) | 0.631 |
| BNP (pg/mL)^*^ | 94.50(81.01-108.48) | 94.03(80.21-108.26) | 98.45(85.93-109.29) | 0.024 |
| APTT (s)^*^ | 35.20(31.70-38.70) | 35.10(31.60-38.65) | 35.30(32.50-38.90) | 0.244 |
| PT (s)^*^ | 15.20(13.70-17.40) | 15.20(13.70-17.40) | 15.30(13.60-17.40) | 0.761 |
| INR^*^ | 1.27(1.10-1.70) | 1.25(1.09-1.60) | 1.46(1.20-3.26) | ＜0.001 |
| Fibrinogen (g/L)^*^ | 4.07(3.70-4.44) | 4.07(3.69-4.43) | 4.11(3.77-4.75) | 0.003 |
| D-dimer (mg/L)^*^ | 2.98(1.65-6.31) | 2.89(1.66-6.26) | 2.85(1.57-7.02) | 0.128 |
| Lactic acid (mmol/L)^*^ | 4.40(3.71-5.12) | 4.38(3.69-5.07) | 4.61(3.91-5.48) | 0.005 |
| Procalcitonin (μg/L)^*^ | 3.03(2.69-3.39) | 3.01(2.67-3.36) | 3.14(2.88-3.53) | ＜0.001 |
| CRP (mg/L)^*^ | 45.30(18.04-88.30) | 42.70(17.19-85.55) | 67.11(30.58-110.00) | ＜0.001 |
| **Treatment measures, *n* (%)** |  |  |  |  |
| Corticosteroid use^§^ | 366(23.3) | 346(24.7) | 20(12.0) | ＜0.001 |
| Epinephrine use^§^ | 96(6.1) | 90(6.4) | 6(3.6) | 0.149 |
| Norepinephrine use^§^ | 383(24.4) | 325(23.2) | 58(34.7) | 0.001 |
| Dopamine use^§^ | 322(20.5) | 270(19.3) | 52(31.1) | ＜0.001 |

*^†^* Normally distributed continuous variables are presented as means with standard deviations and analyzed by Student’ s t-test.

^*^ Non-normally distributed continuous variables are presented as medians with interquartile ranges and analyzed by non-parametric test.

^§^ Categorical variables are presented as frequencies with percentages and analyzed by Chi-square test or Fisher’ s exact test.

MAP, mean arterial pressure; BMI, body mass index; COPD, chronic obstructive pulmonary disease; SOFA score, sequential organ failure assessment score; APACHE Ⅱ score, acute physiology and chronic health evaluation Ⅱ score; SAPS Ⅱ, simplified acute physiology score Ⅱ; ALT, alanine aminotransferase; BNP, B-type natriuretic peptide; APTT, activeated partial thromboplasting time; PT, prothrombin time; INR, international normalized ratio; CRP, C-reaction protein.

**Supplementary Table 3.** Coefficients and lambda.1-SE value of the LASSO regression.

| **Variables** | **Coefficients** | **Lambda.1-SE** | **log(Lambda)** |
| --- | --- | --- | --- |
| Intercept | -5.503 | 0.021 | -3.855 |
| Age (years) | 0.006 |  |  |
| **Comorbidity** |  |  |  |
| Congestive heart failure | 0.027 |  |  |
| **Severity on admission** |  |  |  |
| SOFA score | 0.213 |  |  |
| **Laboratory tests** |  |  |  |
| INR | 0.310 |  |  |
| Fibrinogen (g/L) | 0.154 |  |  |
| CRP (mg/L) | 0.008 |  |  |
| **Treatment measures** |  |  |  |
| Dopamine use | 0.056 |  |  |

SOFA score, sequential organ failure assessment score; INR, international normalized ratio; CRP, C-reaction protein.

**Supplementary Table 4.** Diagnostic performance of the nomogram model for new-onset atrial fibrillation in the training and validation cohorts.

| **Variable** | **Value** | |
| --- | --- | --- |
|  | **Training Cohort** | **Validation Cohort** |
| AUROC | 0.861(0.830-0.892) | 0.845(0.804-0.886) |
| Sensitivity | 0.737 | 0.892 |
| Specificity | 0.844 | 0.687 |
| Cut-off value | 0.108 | 0.076 |
| False positive rate | 0.156 | 0.313 |
| False negative rate | 0.263 | 0.108 |

AUROC, area under the receiver operating characteristic curve.

**Supplementary Table 5.** Comparison of different severity of sepsis in the training cohort.

| **Variables** | **Sepsis**  **(n=875)** | **Severe sepsis**  **(n=448)** | **Septic shock**  **(n=245)** | ***P* value** |
| --- | --- | --- | --- | --- |
| Age (years)*^†^* | 58.43±15.85 | 59.97±16.24 | 60.88±17.39^a^ | 0.062 |
| **Comorbidity, *n* (%)** |  |  |  |  |
| Congestive heart failure^§^ | 206(23.5) | 109(24.3) | 49(20.0) | 0.409 |
| **Severity on admission** |  |  |  |  |
| SOFA score^*^ | 4.00(3.00-7.00) | 6.00(3.00-8.00)^a^ | 6.00(3.00-8.00)^a^ | ＜0.001 |
| **Laboratory tests** |  |  |  |  |
| INR^*^ | 0.81(0.65-0.95) | 0.79(0.66-0.95) | 0.80(0.63-0.94) | 0.613 |
| Fibrinogen (g/L)^*^ | 4.03(3.66-4.40) | 4.10(3.73-4.44)^a^ | 4.16(3.76-4.55)^a^ | 0.003 |
| CRP (mg/L)^*^ | 43.10(17.20-84.70) | 45.95(18.02-90.89) | 61.00(20.75-109.20)^ab^ | 0.004 |
| **Treatment measures, *n* (%)** |  |  |  |  |
| Dopamine use^§^ | 157(17.9) | 97(21.7) | 68(27.8)^ab^ | 0.003 |
| **Outcomes, *n* (%)** |  |  |  |  |
| New-onset atrial fibrillation^§^ | 41(4.7) | 73(16.3)^a^ | 53(21.6)^ab^ | ＜0.001 |
| In-hospital mortality^§^ | 132(15.1) | 105(23.4)^a^ | 79(32.2)^ab^ | ＜0.001 |

*^†^* Normally distributed continuous variables are presented as means with standard deviations and analyzed by Student’ s t-test.

^*^ Non-normally distributed continuous variables are presented as medians with interquartile ranges and analyzed by non-parametric test.

^§^ Categorical variables are presented as frequencies with percentages and analyzed by Chi-square test or Fisher’ s exact test.

SOFA score, sequential organ failure assessment score; INR, international normalized ratio; CRP, C-reaction protein.

^a^ Compared with the sepsis group, *P*＜0.05;

^b^ Compared with the severe sepsis group, *P*＜0.05;

**Supplementary Table 6.** Comparison of different severity of sepsis in the validation cohort.

| **Variables** | **Sepsis**  **(n=517)** | **Severe sepsis**  **(n=239)** | **Septic shock**  **(n=168)** | ***P* value** |
| --- | --- | --- | --- | --- |
| Age (years)*^†^* | 57.48±16.80 | 61.66±17.52^a^ | 64.51±13.87^a^ | ＜0.001 |
| **Comorbidity, *n* (%)** |  |  |  |  |
| Congestive heart failure^§^ | 88(17.0) | 61(25.5)^a^ | 46(27.4)^a^ | 0.003 |
| **Severity on admission** |  |  |  |  |
| SOFA score^*^ | 3.00(2.00-5.00) | 4.00(2.00-6.00) | 4.00(2.00-6.00)^ab^ | ＜0.001 |
| **Laboratory tests** |  |  |  |  |
| INR^*^ | 0.80(0.64-0.93) | 0.81(0.70-0.97) | 0.84(0.68-0.98)^a^ | 0.011 |
| Fibrinogen (g/L)^*^ | 4.01(3.66-4.40) | 4.12(3.69-4.49) | 4.03(3.70-4.49) | 0.109 |
| CRP (mg/L)^*^ | 44.00(16.25-93.90) | 48.10(17.30-97.87) | 48.40(20.17-86.55)^ab^ | 0.001 |
| **Treatment measures, *n* (%)** |  |  |  |  |
| Dopamine use^§^ | 102(19.7) | 63(26.4)^a^ | 51(30.4)^a^ | 0.008 |
| **Outcomes, *n* (%)** |  |  |  |  |
| New-onset atrial fibrillation^§^ | 22(4.3) | 38(15.9)^a^ | 42(25.0)^ab^ | ＜0.001 |
| In-hospital mortality^§^ | 104(20.1) | 60(25.1) | 58(34.5)^ab^ | 0.001 |

*^†^* Normally distributed continuous variables are presented as means with standard deviations and analyzed by Student’ s t-test.

^*^ Non-normally distributed continuous variables are presented as medians with interquartile ranges and analyzed by non-parametric test.

^§^ Categorical variables are presented as frequencies with percentages and analyzed by Chi-square test or Fisher’ s exact test.

SOFA score, sequential organ failure assessment score; INR, international normalized ratio; CRP, C-reaction protein.

^a^ Compared with the sepsis group, *P*＜0.05;

^b^ Compared with the severe sepsis group, *P*＜0.05;

**Supplementary Table 7.** Comparison of AUC results of nomogram model in sepsis of different severity.

| **AUC** | ***P* value** | |
| --- | --- | --- |
|  | **Training Cohort** | **Validation Cohort** |
| AUC _Severe sepsis_ VS. AUC _Sepsis_ | 0.075 | 0.273 |
| AUC _Severe sepsis_ VS. AUC _Septic shock_ | 0.408 | 0.420 |
| AUC _Sepsis_ VS. AUC _Septic shock_ | 0.005 | 0.022 |


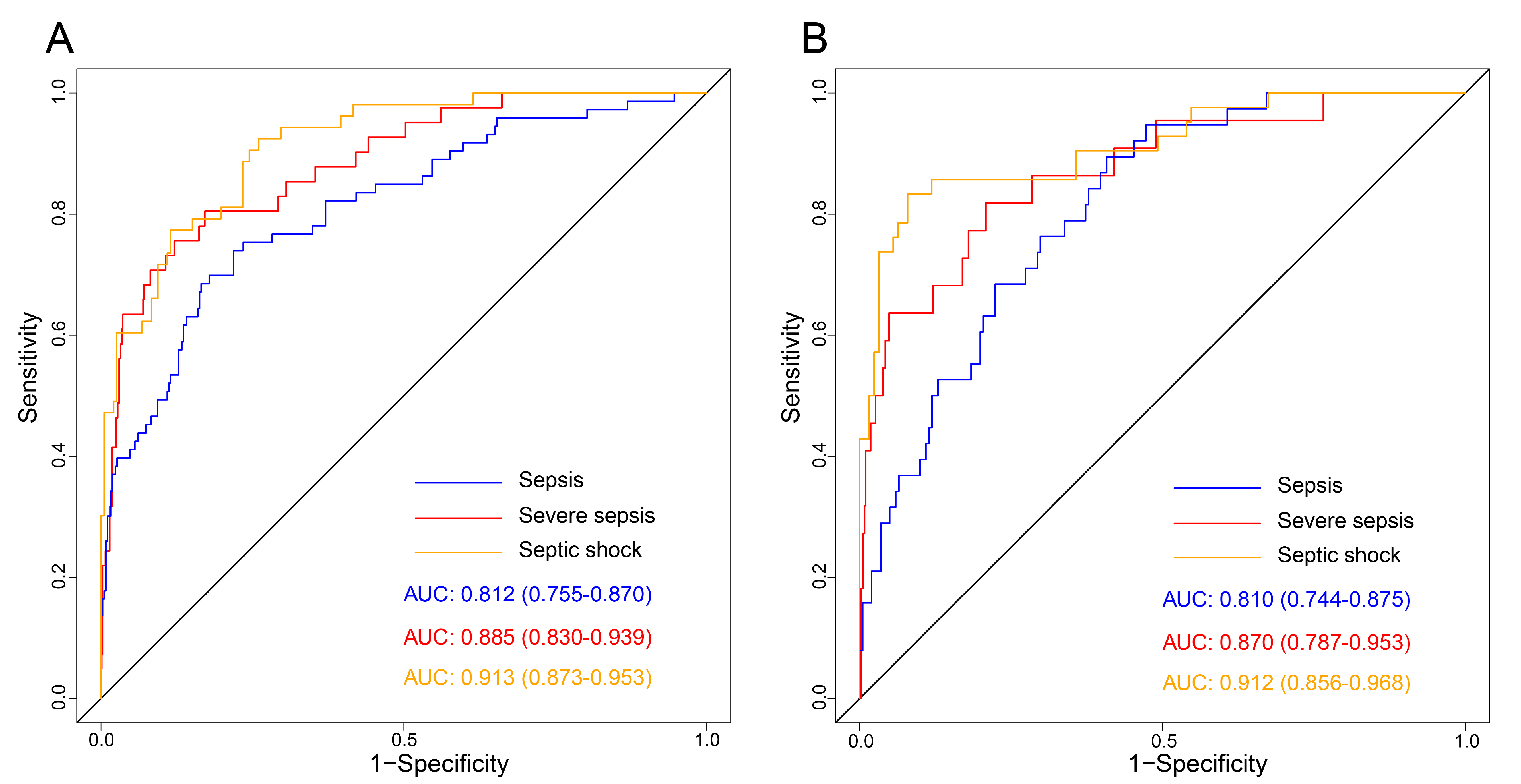


**Supplementary Figure 1.** Comparison of the predictive performance of nomogram model in sepsis of different severity. **(A)** Training cohort; **(B)** Validation cohort.
